# The ‘Tasty School’ model is feasible for food education in primary schools

**DOI:** 10.1111/jhn.13071

**Published:** 2022-08-15

**Authors:** Aija Liisa Laitinen, Amma Antikainen, Santtu Mikkonen, Kaisa Kähkönen, Sanna Talvia, Silja Varjonen, Saila Paavola, Leila Karhunen, Tanja Tilles‐Tirkkonen

**Affiliations:** ^1^ Department of Clinical Nutrition, Institute of Public Health and Clinical Nutrition University of Eastern Finland Kuopio Finland; ^2^ Department of Applied Physics University of Eastern Finland Kuopio Finland; ^3^ Department of Environmental and Biological Sciences University of Eastern Finland Kuopio Finland; ^4^ School of Applied Educational Science and Teacher Education University of Eastern Finland Joensuu Finland; ^5^ Finnish Society for Food Education Ruukku Helsinki Finland

**Keywords:** education models, feasibility, food education, schools

## Abstract

**Background:**

The ‘Tasty School’ is a tailored teacher‐delivered food education model for primary schools in Finland. The aim of the current study was to investigate the feasibility of the Tasty School model in primary schools. Furthermore, the aim was to assess changes during the intervention in the class teachers' perspectives and experiences related to food education and school dining.

**Methods:**

The method involved a quasi‐experimental study with intervention and control groups. A total of 130 class teachers from 15 intervention and 10 control schools from five municipalities in Finland participated in the study during one school year. The theoretical framework of acceptability was utilised to evaluate feasibility using frequencies. The comparison data were analysed using a mixed‐effects model for repeated measures to account for the intervention effects and selected standardising effects.

**Results:**

Teachers reported that the model was highly acceptable and easily integrated into the school environment. Support from principals and colleagues was the most important facilitator of food education, and lack of time was the barrier. Teachers in the intervention schools were more likely to consider school meals healthy after the intervention, and they reported having sufficient materials and supplies for food education.

**Conclusions:**

The Tasty School was shown to be a feasible model for food education in primary schools. The current study especially found that the commitment of the whole school and principals' role are crucial in the implementation of food education. The factors that support the implementation must be strengthened, and efforts must be made to reduce the barriers.

## Key points

- The Tasty School was shown to be a feasible model for food education in primary schools.
- The commitment of the whole school and principal's role are crucial in the implementation of food education.

## Graphical abstract

The ‘Tasty School’ model is feasible for food education in primary schools. The whole school's commitment and the principal's role are crucial in the effective implementation of food education.

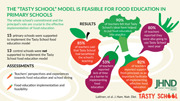

## INTRODUCTION

School is a promising arena for promoting healthy eating patterns because it reaches practically all children in Finland.^1^, ^2^ Moreover, in Finland a free hot meal is offered daily to all pupils in basic education.^3^ The content of the school meals is balanced and healthy because it is based on and guided by the Finnish National Nutrition Council's School Meal Recommendations.^4^ The recommendations also guide food education implemented during school dining, suggesting, for example, the social participation of the pupils and of the teaching staff eating together with the pupils. The Finnish National Core Curriculum for Basic Education^5^ identifies food education as an essential part of school routines and obligates schools to set objectives for it. However, the curriculum does not provide practical tools or more specific guidance for the implementation of food education in practice.

An earlier study showed that a food education curriculum called ‘Tools for Feeling Good’ was effective in promoting regular meals, vegetable consumption and eating varied school lunches among fifth graders.^6^ Effective food education models have been developed, but establishing food education in normal everyday school practices is challenging.^7^, ^8^ Several nutrition and food education interventions have been structured as separate projects apart from the normal routine of primary schools.^9^, ^10^, ^11^

The ‘Tasty School’ model provides tools to implement food education in primary schools and thus helps schools to meet the requirements of the School Meal Recommendations^4^ and Finnish National Curriculum.^5^ The Tasty School model was developed in cooperation with primary schools and nutrition, food education and basic education experts by utilising a previous nutrition curriculum, ‘Tools for Feeling Good’, as a starting point.^6^ The food education model extended the earlier‐developed curriculum to cover the entire primary school system (grades 1–6, age 7–12 years) and diversified through a website that included an idea bank with learning materials, self‐assessment questionnaire and online training for teachers. The model was based on several theoretical approaches: self‐determination theory,^12^ health at every size approach,^13^ eating competence,^14^ sensory‐based learning^15^ (i.e., Sapere), mindful eating^16^ and intuitive eating.^17^ The Tasty School has a holistic approach as it integrates food education pedagogy in school subjects, school meals and school environment. At the teachers' level, the aim of the Tasty School model was to increase their knowledge and pedagogical competence in food education through online training and to provide a wide set of tools for evaluating, planning and implementing food education in primary schools.

Our intention was that the Tasty School model would become embedded in both curriculum development and lesson planning, thereby ensuring that food education occurs in the classroom as well as the cafeteria and, furthermore, designating school meals as a pedagogical activity.^18^ It is important to understand the elements of effective implementation to establish food education pedagogy in the daily routines of primary schools.^19^, ^20^ Thus, we need a broader picture of the Tasty School model's feasibility to promote its establishment, because it is a new, strongly teacher‐driven and applied food education model.

The aim of the current study was to investigate the feasibility of the Tasty School food education model in primary schools. Furthermore, the aim was to assess changes during the intervention in the class teachers' perspectives and experiences related to food education and school dining.

## METHODS

The present study had a quasi‐experimental design^21^ with 15 intervention schools that were supported to implement food education based on the Tasty School model and 13 control schools that did not receive food education support. The study was conducted during the 2019–2020 school year and included baseline and follow‐up questionnaires addressed to all class teachers in participating schools. Also, feasibility questionnaire was addressed to teachers in the intervention group after the intervention. The study design was reviewed and approved by the Committee on Research Ethics of the University of Eastern Finland.

### Participants and recruitment

The recruitment process started in January 2019 by contacting the municipalities' directors of education and inviting them to participate in the study. Based on their previous cooperation, six municipalities were contacted, and five municipalities located in southern and eastern Finland finally participated in the study. These municipalities had approximately 10,000–120,000 inhabitants and 83 primary schools.

Schools from participating municipalities were recruited in spring 2019 by the directors of education. The aim was to recruit a maximum of 15 intervention and 15 control schools due to the project's resources. Due to the varied resources of schools, the participating schools were able to choose their status in the study, as either an intervention school or a control school. The directors of education were not willing to order schools to participate, and thus, all forms of participation were known up front. At the end of the recruiting process when the maximum number of intervention schools was already recruited, the rest of the interested schools were invited to participate as control schools. At least one control school was recruited from each participating municipality.

**Figure 1:**
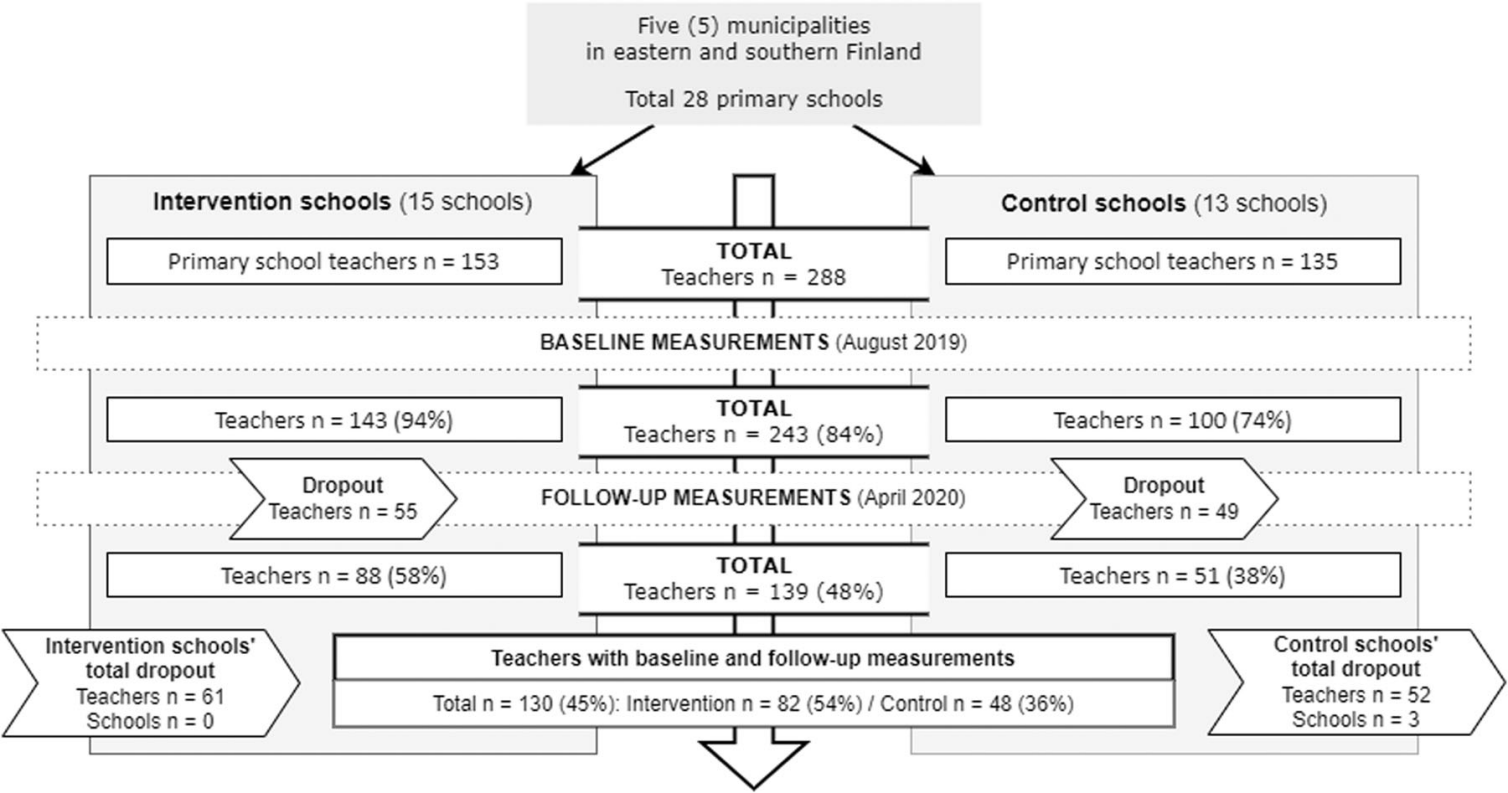
Study design, study population and measurements

Altogether 15 intervention and 13 control schools participated in the study (Figure 1). Each municipality had at least four participating schools, and all primary schools participated in one municipality. All class teachers in the intervention and control schools were asked to participate, and all of them provided a written consent to participate in the study.

### Implementation of the Tasty School model

The study group introduced the Tasty School model in spring 2019 to the teachers of the intervention schools. All intervention schools were advised to implement a tailored, teacher‐delivered Tasty School programme during the school year 2019–2020 from September 2019 to March 2020.

**Figure 2:**
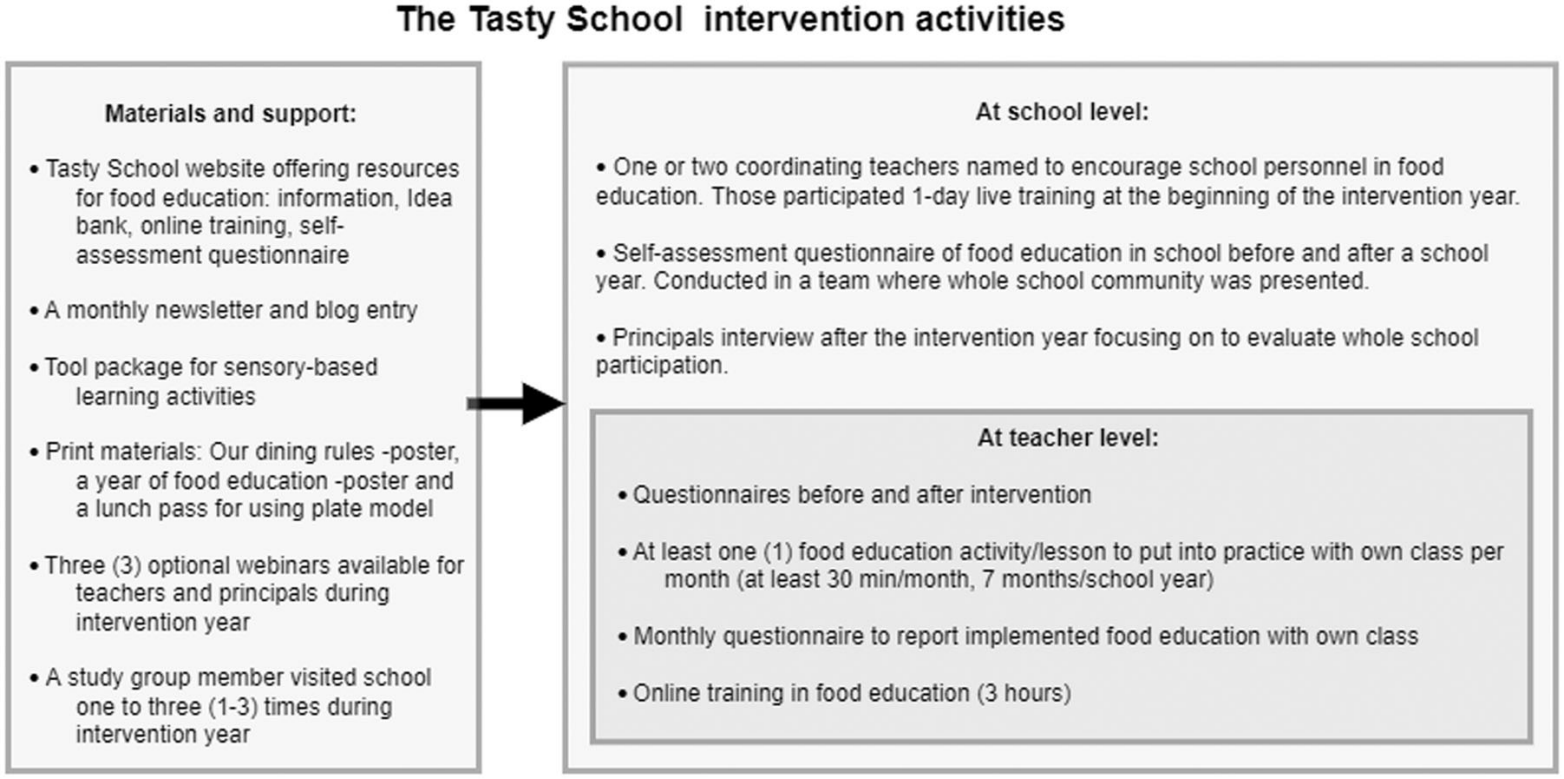
The Tasty School intervention activities at the teacher and school levels and the materials and support provided for the intervention schools

Figure 2 describes the Tasty School intervention activities at the teacher and school levels and the support and materials provided for intervention schools. For each intervention school the starting point of the implementation was to fill in a self‐assessment questionnaire at the beginning of the school year concerning the state of their school's food education and school lunch arrangements. The survey covered five themes: management and engagement, integration of food education, implementation of school meals, collaboration and support. The questionnaire was filled by a multi‐professional group. Schools were instructed to invite at least the principal, a teacher and a food service employee into the multi‐professional group, but others were also welcomed to participate. Based on the self‐assessment questionnaire, each intervention school was advised to choose independently development targets to guide the implementation of the Tasty School model at school level. The self‐assessment questionnaire was used only for assessing the current state of food education and setting targets for schools. The information gathered from it was not used as research data.

The schools selected one to two coordinating teachers who encouraged school personnel to implement the Tasty School programme. These coordinating teachers received a 1‐day live training on the Tasty School at the beginning of the school year. A member of the study group contacted the coordinating teachers once in a month by email or phone throughout the school year. Each class teacher was instructed to implement at least one food education idea (duration of at least 30 min) monthly in their class (seven ideas per school year). Class teachers were also encouraged to conduct a 3‐h online training on food education during the autumn semester.

Teachers were instructed to utilise the Tasty School idea bank, which contained more than 100 development or action ideas for food education in school. These ideas were instructed to be integrated into their schools’ daily routines during the intervention year. The idea bank is available at www.maistuvakoulu.fi (only in Finnish). The idea bank had three main sections: ideas for lessons, school dining and collaboration in food education. The ideas were further grouped by topics, such as food culture, food routes, sensory‐based learning (Sapere), media literacy, sustainable diets, body image and nutrition and health.

During the intervention a monthly newsletter with practical ideas for food education was emailed to all teachers. The Tasty School web page published a monthly food education blog entry with topical issues and tips. Weekly food education tips were posted on the Tasty School Facebook and Instagram pages. Schools received a copy of the Finnish Recommendations for School Meals and a toolkit for sensory‐based food education activities (e.g., smelling bottles, basic tastes in white powder form and bags for touching with printed handbook). The schools received no financial support.

### Assessments

#### Evaluation of implementation and feasibility

Implementation fidelity, referring to the degree to which the intervention was delivered as intended,^22^ was evaluated monthly during the intervention via electronic questionnaires targeted only at the intervention group. Class teachers reported the quantity and nature of food education activities they had conducted and the time spent on food education. Through the questionnaires, teachers reported how many and what kinds of activities they had completed during each month.

Feasibility, including acceptability and implementation fidelity of the Tasty School model, was also evaluated. A feasibility questionnaire was directed to the teachers after the intervention to investigate how well suited the model is to the school routines and work of teachers. The theoretical framework of acceptability, comprising seven components (affective attitude, burden, perceived effectiveness, ethicality, intervention coherence, opportunity costs and self‐efficacy), was utilised.^23^ In addition, implementation barriers and facilitators were evaluated with a query that included 16 statements. All questions were asked using a five‐point Likert‐scale: totally disagree, somewhat disagree, neither agree nor disagree, somewhat agree and totally agree, scored 1, 2, 3, 4 and 5. The feasibility questionnaire was pre‐tested before it was implemented. About 20 people participated in the pre‐testing.

All principals (*n* = 15) of the intervention and control schools (*n* = 10) were personally interviewed by phone after the intervention in June or October 2020. In the interviews principals were asked (1) whether the Tasty School model was included in the school year plan, (2) whether food education projects in which the entire school participated were implemented during the previous school year, (3) whether any positive changes were noticed at school (subjective evaluation) and (4) whether the school intended to take advantage of the Tasty School model next school year. Also, an opportunity to give open feedback was provided.

#### Teachers' perspectives and experiences of food education and school dining

All class teachers in the intervention and control schools were invited to answer an electronic baseline questionnaire at the beginning of the school year in August 2019 and an electronic follow‐up questionnaire in April 2020. Three control schools dropped out due to the COVID‐19 pandemic before the follow‐up measures. Supporting Information, Table 1, presents descriptive information on the participating teachers.

The perspectives and experiences on both food education and school dining were evaluated using 15 statements. The statements in the school dining query were based on a previously used questionnaire^24^ (Supporting Information, Table 2). These statements were evaluated using a five‐point Likert scale: totally disagree, somewhat disagree, neither agree nor disagree, somewhat agree and totally agree, scored 1, 2, 3, 4 and 5. The food education query, developed by the study group, included the following themes: ‘resources available for food education’ (financial resources, time and materials), ‘support’, ‘self‐efficacy concerning food education as a part of school teaching’, ‘importance of food education at school and as a part of teacher's work’ and ‘effects of food education on pupil's health and well‐being’.

### Statistical analysis

Statistical data analysis was performed using SPSS (IBM SPSS, version 27.0, 2020). Descriptive statistics (means, standard deviations and frequencies) were calculated separately for baseline, follow‐up and feasibility questionnaires. More complex statistical analysis was enrolled for comparing changes in teachers' perspectives and experiences between intervention and control schools. The criterion for significance was set to be *p* < 0.05. Associations with statement variables, measured using the five‐point Likert scale, and explanatory variables were analysed using linear mixed‐effects models for repeated measures due to a two‐level data structure by clustering the repeated outcome measures at baseline and follow‐up within teachers. Intervention versus control was examined (two groups). The model was adjusted for fixed effects of sex and teaching experience and included main effects for time and two‐level intervention group with intervention group × time interaction. The only random effect in the model, in addition to repeated structure, was the teacher‐specific intercept. Clustering within schools was tested in the analysis, but it did not have significant effect on the results, and thus, in the means of model simplicity, it was excluded from the final model. Normality and independence of residuals, assumed by the mixed model, were confirmed with visual inspection of histograms and autocorrelation plots of the residual, respectively.

### Dropout analysis

Dropout analysis was conducted using the Mann–Whitney *U‐*test to examine possible selection bias between teachers (*n* = 115) who dropped out after the baseline and teachers (*n* = 130) who remained in the study. No significant differences were found in any of the descriptive or outcome variables.

## RESULTS

### Feasibility evaluation through acceptability and implementation fidelity

There were 153 class teachers in the intervention schools. Teachers implemented an average of three food education activities during the school year (mean 3.0, standard deviation 2.2, min 0, max 7). A total of 11% (*n* = 17) of the teachers never answered the monthly report or did not implement any food education activities during school year.

After the intervention, 88 teachers (58%) in the intervention schools answered the feasibility questionnaire concerning user experiences and perceptions of feasibility and acceptability of the model. Online training was fully completed by 60% (*n* = 53) and partially completed by 15% (*n* = 13). Overall 96% (*n* = 84) reported they had utilised the Tasty School's food education idea bank during the school year. The Tasty School's feasibility and acceptability was high: 80% (*n* = 70) of teachers reported they were also going to use the Tasty School next year, 80% (*n* = 70) would recommend the model to colleagues, 90% (*n* = 79) felt that Tasty School's ideas have helped to put food education into practice and 85% (*n* = 74) said that Tasty School had benefitted the school's teaching (Supporting Information, Table 3). One‐fourth (25%, *n* = 22) of the teachers felt that the Tasty School has increased cooperation with pupils’ parents. Principals' interviews indicated that 80% of the intervention schools aimed to continue implementing the Tasty School next year.

### Facilitators for and barriers to implementing Tasty School model

**Figure 3:**
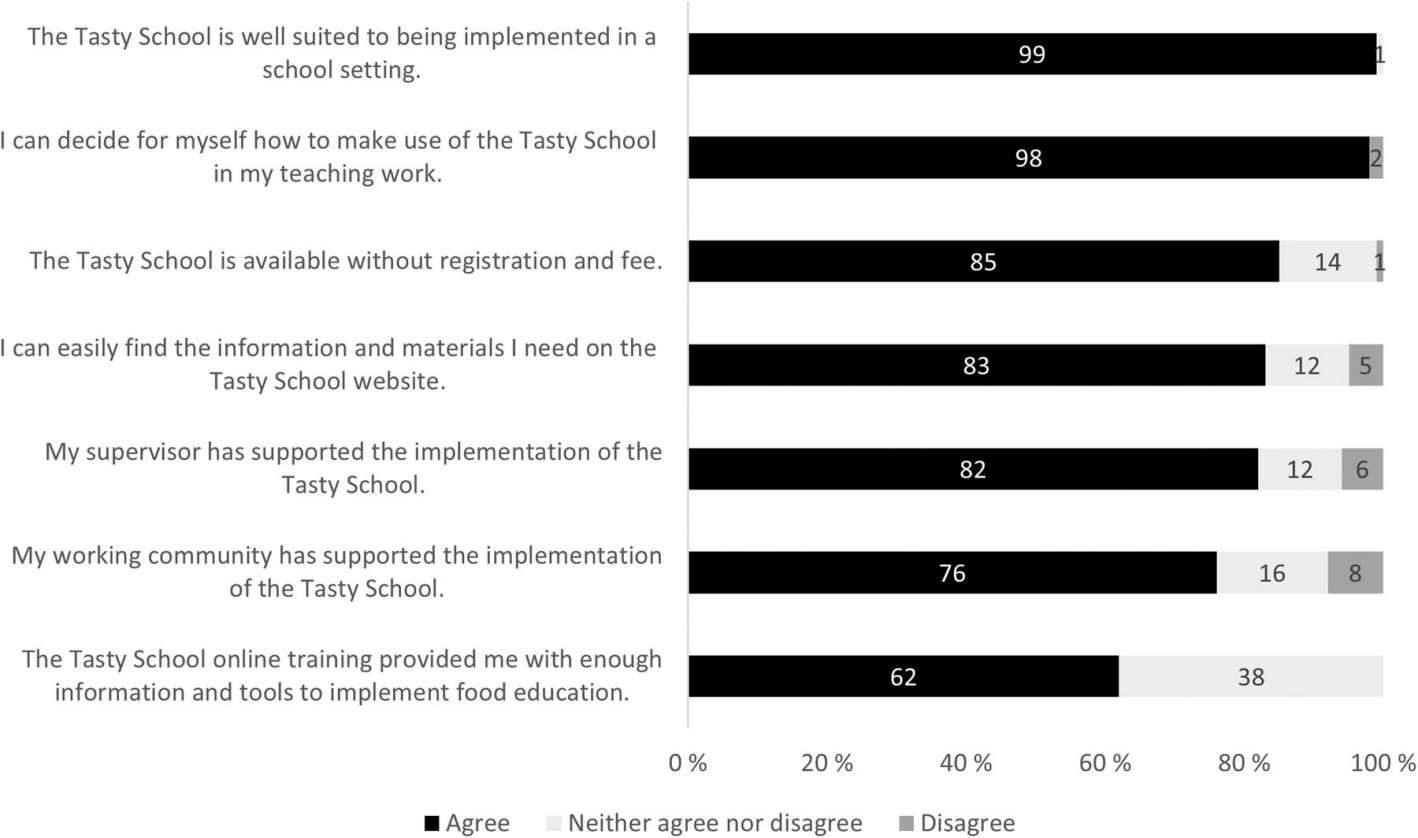
Facilitators for implementing the Tasty School model according to the experiences of class teachers

**Figure 4:**
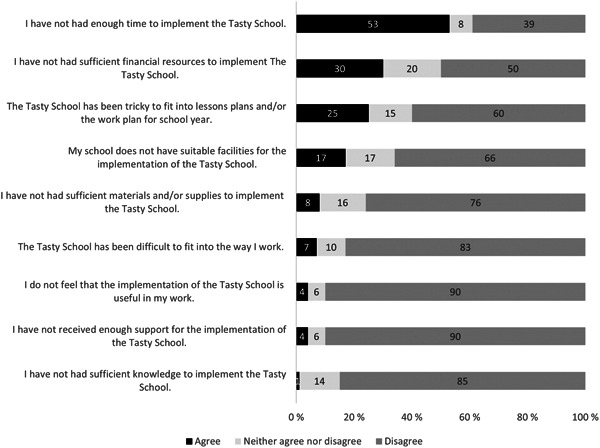
Barriers to implementing the Tasty School model according to the experiences of class teachers

The most important implementation facilitators were support from principals and colleagues, and the Tasty School can be used based on the teachers' own needs and interests (Figure 3). The model was also reported to be highly suitable for the school environment: only 1% (*n* = 1) of the teachers neither agree nor disagree that the Tasty School model is appropriate for the school setting. The reported barriers (Figure 4) were the lack of time (53%, *n* = 47, of teachers), financial resources (30%, *n* = 26, of teachers), difficulties in work planning (25%, *n* = 22, of teachers) and suitable room/space for food education (17%, *n* = 15, of teachers).

### Comparison of teachers' perspectives and experiences between the intervention and control groups

The study sample comprised 130 teachers from intervention and control schools who had answered questionnaires both at baseline and at follow‐up (Figure 1).

### Food education

At baseline, 92% (*n* = 119) of teachers agreed that the school has an important role in their pupils' food education, and 89% (*n* = 116) of teachers agreed that class teachers are important food educators for pupils. Teachers in the intervention schools (*n* = 82) experienced an increase in their resources for implementing food education during the intervention (Table 1). The teachers reported that they received greater support from the work community and had sufficient learning materials and supplies to implement food education at the end of the intervention. Furthermore, they agreed that food education can affect their pupils’ well‐being. More modest or even opposite changes were observed in the control group (*n* = 48).

**Table 1:** Statistical comparison of teachers' perspectives and experiences of food education

Statement^a^ (min 1, max 5)	Intervention group (*n* = 82)	Control group (*n* = 48)	*p*‐Value^b^
*The school has an important role in food education of pupils*			
Mean at baseline (SD)	4.35 (0.71)	4.19 (0.76)	0.908
Mean at follow‐up (SD)	4.40 (0.54)	4.23 (0.81)	
*Class teachers are important food educators for pupils*			
Mean at baseline (SD)	4.30 (0.70)	4.04 (0.77)	0.874
Mean at follow‐up (SD)	4.35 (0.61)	4.13 (0.89)	
*Food education can affect pupils' well‐being*			
Mean at baseline (SD)	4.40 (0.65)	4.44 (0.62)	**0.017**
Mean at follow‐up (SD)	4.57 (0.52)	4.27 (0.82)	
*Food education can affect well‐being by promoting pupils' healthy eating habits*			
Mean at baseline (SD)	4.46 (0.53)	4.38 (0.64)	0.608
Mean at follow‐up (SD)	4.39 (0.56)	4.23 (0.83)	
*Food education is an important part of my work*			
Mean at baseline (SD)	3.85 (1.00)	3.90 (0.90)	0.129
Mean at follow‐up (SD)	3.99 (0.82)	3.75 (1.00)	
*I am interested in developing food education in (my) school*			
Mean at baseline (SD)	3.91 (0.89)	3.52 (1.01)	0.472
Mean at follow‐up (SD)	3.70 (0.89)	3.42 (1.09)	
*I feel/believe that I can make an impact on my pupils’ well‐being by food education*			
Mean at baseline (SD)	3.74 (0.78)	3.50 (1.11)	0.459
Mean at follow‐up (SD)	3.79 (0.68)	3.67 (0.91)	
*I have experienced that I can affect well‐being by promoting pupils’ eating habits with food education*			
Mean at baseline (SD)	3.65 (0.88)	3.58 (0.96)	0.610
Mean at follow‐up (SD)	3.76 (0.73)	3.60 (0.92)	
*I trust that I have sufficient competence to implement food education*			
Mean at baseline (SD)	3.96 (0.76)	4.10 (0.88)	0.212
Mean at follow‐up (SD)	4.17 (0.70)	4.13 (0.79)	
*I feel/believe that I can implement food education as part of my job*			
Mean at baseline (SD)	3.74 (0.89)	3.81 (0.92)	0.293
Mean at follow‐up (SD)	3.91 (0.79)	3.79 (0.90)	
*I have sufficient learning materials and exercises to implement food education*			
Mean at baseline (SD)	2.90 (1.17)	3.06 (1.17)	**<0.001**
Mean at follow‐up (SD)	4.26 (0.68)	3.27 (1.09)	
*I have sufficient supplies to implement food education*			
Mean at baseline (SD)	2.51 (1.11)	2.71 (1.15)	**<0.001**
Mean at follow‐up (SD)	3.45 (1.06)	2.90 (1.06)	
*It is possible for me to obtain paid material for the implementation of food education*			
Mean at baseline (SD)	2.26 (1.02)	2.08 (0.92)	0.562
Mean at follow‐up (SD)	2.18 (1.03)	2.12 (0.98)	
*I have sufficient time to implement food education*			
Mean at baseline (SD)	2.48 (1.10)	2.52 (1.07)	0.500
Mean at follow‐up (SD)	2.49 (1.10)	2.67 (1.02)	
*I receive sufficient support for my food education work in my work community*			
Mean at baseline (SD)	3.18 (0.90)	3.31 (0.99)	**<0.001**
Mean at follow‐up (SD)	3.83 (0.81)	3.13 (0.91)	

Abbreviation: SD, standard deviation.

^a^ Perspectives on food education at school were evaluated using a 15‐item query with a five‐point Likert‐scale: totally disagree, somewhat disagree, neither agree nor disagree, somewhat agree and totally agree.

^b^ *p*‐Value of the interaction. The data were analysed using a mixed‐effects model for repeated measures accounting for the intervention effect and selected standardising effects.

### School dining

At baseline, 71% (*n* = 91) of teachers agreed that school meals are tasty, 70% (*n* = 90) of teachers agreed that school lunch is a nice moment in a day and 61% (*n* = 79) of teachers agreed that it is cosy in the dining hall, but 57% (*n* = 73) of teachers disagreed that they have enough time to eat, and only 24% (*n* = 31) of teachers agreed that there is not too much noise in the dining hall. Teachers reported that they have 14 min to eat school lunch (*n* = 127, mean: 13.6 min, standard deviation: 4.3, min: 5 and max: 30). The reported time does not include the time it takes to queue and take food.

The teachers' experience participating in planning school dining was low; 63% (*n* = 52) of teachers in the intervention group and 69% (*n* = 33) of teachers in the control group disagreed with the statement, ‘I get to participate in planning school dining’.

The opinion that school meals are healthy strengthened (*p* = 0.012, mean before 4.25 and mean after 4.33) in the intervention schools after the intervention, whereas this opinion weakened (mean before 4.23 and mean after 4.02) in the control group. There were no other statistically significant changes between the teachers of intervention and control schools in their perspectives and experiences of school dining (Supporting Information, Table 2).

## DISCUSSION

Research data on food education embedded in normal school operation are limited. The current study implemented the Tasty School model that focused on integrating food education into the school curriculum, culture and daily pedagogical activity. The current study has a pioneering role because of the nature of the implemented model. The model sought to promote a holistic approach to food education with diverse themes and perspectives.

The feasibility of the Tasty School model was high among the teachers. The model was developed in collaboration with teachers, which has likely increased engagement and feasibility.^25^, ^26^ A further reason for its high feasibility may be that the teachers were able to implement the model according to the needs of their class or school.^27^ According to self‐determination theory,^12^ the model emphasised teacher autonomy and applicability to different kinds of schools and teachers.

The most important implementation facilitator perceived was support from the principals and colleagues. A previous study from the United States reported similarly that teachers need support from school principals to implement nutrition education.^28^ The implementation of the Tasty School model increased the experienced support from work community for food education. It is important that the whole school staff is committed to the implementation of food education, including the principal, who seems to have a crucial role. The themes of food education are diverse and connect to different subjects, including school dining and overall well‐being and sustainability.^29^ Thus, food education cannot be effective if only some of the teachers are interested and involved.

The major barriers to implementing the Tasty School model were lack of time and financial resources and difficulties in work planning. Previous studies have identified similar kinds of barriers for school food education, including teachers' heavy workload, lack of a universal and systematic model or curriculum, lack of common objectives of food education and lack of continuous nutrition training for teachers.^20^, ^30^ The current study shows that limited opportunities for teachers to participate in planning school dining also are a clear challenge for enhancing pupils' social participation in school dining. Defining objectives for food education, active planning and implementation are needed to overcome these difficulties and to achieve sustaining positive effects and efficiency. One way to increase teachers' activity in food education in the future may be to strengthen teachers' views on the pedagogical possibilities of school dining through education, national regulations and guidelines.^18^

At baseline, the teachers in both the intervention and control schools already considered the role of food education in schools important. After the intervention, teachers in the intervention group felt more often that the well‐being of pupils can be influenced by food education, whereas this conviction weakened in the control group. The Tasty School model provided teachers’ sufficient supplies, learning materials and exercises to implement food education. A favourable change can be observed although the amount of implemented food education activities did not fulfil the original aim and up to 40% of the teachers in the intervention schools did not complete the online training. This significant change also emerged even though intervention schools received no financial support, only the Tasty School website and a toolkit for sensory‐based food education. The findings of the present study suggest that the Tasty School model can increase teachers' knowledge and pedagogical competence in food education, and more active participation could further support Tasty School's implementation.^31^

The opinion that school meals are healthy decreased in the control group, whereas the intervention strengthened this view in those teachers. Finnish school meals commonly include everyday dishes, and their nutritional quality is guided by the School Meal Recommendations,^4^ which are based on the Finnish Dietary Recommendations.^32^ Thus, school meals can objectively be kept healthy. Increased opinions school meals being healthy, might reflect also increased positive attitude towards school dining overall. One might also question whether teachers' nutritional knowledge has increased.^33^

The implementation of the model did not focus on increasing cooperation between school personnel and pupils' parents; neither was the amount of cooperation measured in the current study. Involving parents more could support food education and thus further enhance well‐being, nutrition and food literacy not only in the school environment but also in the home environment.^31^, ^34^ This is one aspect that the Tasty School model could further develop in the future.

A strength of the current study was its intervention–control setting. The research data were also collected extensively across Finland; thus, the results do not feature only a particular area. Furthermore, the research schools varied in sizes and operated in several municipalities, so the results are not the result of an individual municipality's resources or curriculum.

The study has a few limitations. As a quasi‐experimental study, the study frame might be exposed to selection bias. Participating schools could choose whether they want to participate as an intervention or control school; thus, schools were not randomised into the research groups.

The intervention study was conducted in the school year 2019–2020 during which the COVID‐19 pandemic emerged and forced schools to shift into remote teaching 1 month before the follow‐up measurements. This might have impacted teachers' resources and thus the implementation intensity of food education. However, the intervention–control study design likely alleviated these possible effects. The COVID‐19 pandemic also affected the dropout rates, especially in the control group but less in the intervention group, which could reflect their higher commitment to the study. Unfortunately, the pandemic influenced so that 42% of teachers in the intervention group did not answer the feasibility questionnaire. Because the response rate at follow‐up was quite low, it is therefore possible that teachers with the most positive attitudes towards the Tasty School model answered the follow‐up and feasibility questionnaire. However, the baseline dropout analysis found no significant differences between the teachers who dropped out and those who completed the study.

In conclusion, the Tasty School food education model offers a promising tool to primary schools in Finland. The elements of the model could also be applied in other countries. Collaboration with teachers in the development of the Tasty School is likely to enhance its feasibility and teachers' commitment to implement it. To promote the implementation of food education, factors that support the implementation, like commitment of the whole school and suitable premises as well as financial resources, must be strengthened, and efforts must be made to reduce the barriers. Teachers also need direction and support from principals to implement food education. The current study especially shows that the commitment of the whole school and principal's role are crucial and that future research should focus on identifying the facilitators for and barriers to implementing food education in primary schools, particularly from the view of principals and education administration.

## AUTHOR CONTRIBUTIONS

Tanja Tilles‐Tirkkonen, Amma Antikainen, Kaisa Kähkönen, Sanna Talvia, Leila Karhunen and Aija Liisa Laitinen were responsible for study conceptualisation and methodology. Amma Antikainen, Aija Liisa Laitinen and Tanja Tilles‐Tirkkonen were responsible for data curation. Amma Antikainen, Kaisa Kähkönen, Silja Varjonen, Saila Paavola and Tanja Tilles‐Tirkkonen were responsible for investigation and data collection. Santtu Mikkonen conceived the concept for the analysis. Santtu Mikkonen, Amma Antikainen and Aija Liisa Laitinen conducted statistical analysis. Aija Liisa Laitinen and Amma Antikainen are the principal authors of the manuscript. Aija Liisa Laitinen, Amma Antikainen and Tanja Tilles‐Tirkkonen were responsible for preparing the original draft. Santtu Mikkonen, Kaisa Kähkönen, Sanna Talvia, Silja Varjonen, Saila Paavola and Leila Karhunen were responsible for reviewing and editing the manuscript. Tanja Tilles‐Tirkkonen was responsible for supervision and project administration. All authors have read and agreed to the published version of the manuscript.

## CONFLICT OF INTEREST

The authors declare no conflict of interest.

## TRANSPARENT PEER REVIEW

The lead author affirms that this manuscript is an honest, accurate and transparent account of the study being reported. The reporting of this work is compliant with STROBE. The lead author affirms that no important aspects of the study have been omitted and that any discrepancies from the study as planned have been explained.

## ETHICS STATEMENT

The study design was reviewed and approved by the Committee on Research Ethics of the University of Eastern Finland.

## Supporting information

Supplementary information.

Supplementary information.

Supplementary information.
