# Supplementary material for: The ‘Tasty School’ model is feasible for food education in primary schools
Source: J Hum Nutr Diet. 2022 Aug 15;36(1):75–85. doi: 10.1111/jhn.13071 (PMC10087126; doi:10.1111/jhn.13071)
Supplement: Supplementary file 1 — Supplementary information. [file JHN-36-75-s003.docx]

Supplement Table 1. Descriptive Information of Teachers and Schools at Baseline.

| **Descriptive** | **Intervention group**  n = 82 | **Control group**  n = 48 | ***P* value** |
| --- | --- | --- | --- |
|  |  |  |  |
| **Age**  Mean (SD) | years  47 (9)  min 25, max 62 | years  47 (8)  min 30, max 62 | 0.540^b^ |
| **Gender**  Female  Male | percentage / frequency  79% / 65  21% / 17 | percentage / frequency  75% / 36  25% / 12 | 0.573^c^ |
| **Years working as  a class teacher**  Mean (SD) | years  20 (11)  min 1, max 57 | years  17 (9)  min 2, max 38 | 0.313^b^ |
| **Having a school lunch with pupils**  Every day  3-4 times a week  1-2 times a week  Less than once a week  Never | percentage / frequency  85% / 70  12% / 10  1% / 1  0% / 0  1% / 1 | percentage / frequency  92% / 43  6% / 3  0% / 0  2% / 1  0% / 0 | 0.321^d^ |
| **Number of teachers in a school**  Mean | 10  min 3, max 22 | 10  min 3, max 18 |  |
| **Number of pupils in a school**  Mean^a^ | 192  min 51, max 424 | 205  min 50, max 400 |  |

SD=Standard deviation
^a^ The average number of pupils in Finland was 169 in a primary school in 2019. Source: Official statistics of Finland (OSF): Providers of education and educational institutions. Helsinki: *Statistics Finland*. 2019.

^b^ The data were analyzed with Independent Samples T-test.
^c^ The data was analyzed with Chi-Square test.
^d^ The data were analyzed with Kruskal-Wallis test.
