# Supplementary material for: The ‘Tasty School’ model is feasible for food education in primary schools
Source: J Hum Nutr Diet. 2022 Aug 15;36(1):75–85. doi: 10.1111/jhn.13071 (PMC10087126; doi:10.1111/jhn.13071)
Supplement: Supplementary file 2 — Supplementary information. [file JHN-36-75-s002.docx]

Supplement Table 2. Teachers’ Perspectives and Experiences of School Dining. Questionnaire Edited from the Study of Nutrition and Well-being of Secondary School Pupils^24^.

| **Statement^a^**  (min 1, max 5) | **Intervention group**  n = 82 | **Control group**  n = 48 | ***P* value^b^** |
| --- | --- | --- | --- |
| **The personnel of the food service are friendly.**  mean at baseline (SD)  mean at follow-up (SD) | 4.30 (0.91)  4.33 (0.93) | 4.21 (1.15)  4.25 (1.12) | 0.932 |
| **It is peaceful in the dining hall.**  mean at baseline (SD)  mean at follow-up (SD) | 2.58 (1.05)  2.99 (1.11) | 2.58 (1.11)  2.90 (1.02) | 0.622 |
| **There is not too much noise in the dining hall.**  mean at baseline (SD)  mean at follow-up (SD) | 2.30 (1.08)  2.67 (1.09) | 2.42 (1.20)  2.67 (1.04) | 0.572 |
| **It is cozy in the dining hall.**  mean at baseline (SD)  mean at follow-up (SD) | 3.42 (1.01)  3.49 (0.89) | 3.44 (1.03)  3.54 (0.94) | 0.748 |
| **The food queue runs smoothly.**  mean at baseline (SD)  mean at follow-up (SD) | 3.79 (0.86)  3.76 (1.00) | 3.60 (0.94)  3.65 (0.93) | 0.625 |
| **School lunch is a nice moment in a day.**  mean at baseline (SD)  mean at follow-up (SD) | 3.69 (1.01)  3.95 (0.90) | 3.81 (0.94)  4.02 (0.76) | 0.828 |
| **Teachers guide dining appropriately.**  mean at baseline (SD)  mean at follow-up (SD) | 3.96 (0.86)  4.01 (0.76) | 3.98 (0.84)  3.96 (0.82) | 0.559 |
| **Teachers act as a role model for pupils in school dining.**  mean at baseline (SD)  mean at follow-up (SD) | 4.42 (0.76)  4.48 (0.61) | 4.38 (0.70)  4.42 (0.71) | 0.814 |
| **Pupils have enough time to eat.**  mean at baseline (SD)  mean at follow-up (SD) | 4.06 (1.18)  4.05 (1.14) | 4.31 (0.90)  4.44 (0.90) | 0.425 |
| **Teachers have enough time to eat.**  mean at baseline (SD)  mean at follow-up (SD) | 2.59 (1.28)  2.74 (1.23) | 2.96 (1.25)  2.94 (1.34) | 0.458 |
| **School meals are healthy.**  mean at baseline (SD)  mean at follow-up (SD) | 4.25 (0.80)  4.33 (0.77) | 4.23 (0.59)  4.02 (0.86) | **0.012** |
| **School meals are tasty.**  mean at baseline (SD)  mean at follow-up (SD) | 3.75 (0.89)  3.91 (0.79) | 3.62 (0.98)  3.67 (0.88) | 0.395 |
| **School meals looks good.**  mean at baseline (SD)  mean at follow-up (SD) | 3.62 (0.90)  3.74 (0.89) | 3.60 (0.92)  3.71 (0.82) | 0.941 |
| **School meal helps pupils to stay healthy.**  mean at baseline (SD)  mean at follow-up (SD) | 4.28 (0.75)  4.45 (0.55) | 4.23 (0.69)  4.33 (0.69) | 0.634 |
| **School meal helps pupils to manage.**  mean at baseline (SD)  mean at follow-up (SD) | 4.52 (0.74)  4.66 (0.59) | 4.67 (0.52)  4.56 (0.58) | 0.081 |

SD=Standard deviation
^a^ Perspectives and experiences of school dining was evaluated using a 15-item query with a five-point Likert-scale: totally disagree, somewhat disagree, neither agree nor disagree, somewhat agree, totally agree.
^b^ *P* value of the interaction. The data were analyzed with a mixed effects model for repeated measures accounting for the intervention effect and selected standardizing effects.
