# Supplementary material for: The ‘Tasty School’ model is feasible for food education in primary schools
Source: J Hum Nutr Diet. 2022 Aug 15;36(1):75–85. doi: 10.1111/jhn.13071 (PMC10087126; doi:10.1111/jhn.13071)
Supplement: Supplementary file 3 — Supplementary information. [file JHN-36-75-s001.docx]

Supplement Table 3. Experiences of Class Teachers (n = 88) About the Feasibility and Acceptability of the Tasty School Organized According to Seven Components Introduced in Theoretical Framework of Acceptability (TFA).

| Statement | %^a^ | | |
| --- | --- | --- | --- |
|  | **Disagree** | **Neither agree nor disagree** | **Agree** |
| Ethicality  The Tasty School is very much in line with my own values as a teacher. | 0 | 8 | 92 |
| Affective Attitude  I am satisfied that the Tasty School was carried out in my school. | 6 | 13 | 81 |
| I am going to make good use of the Tasty School in my school teaching next school year. | 5 | 15 | 80 |
| I would recommend the Tasty School to my colleagues. | 2 | 18 | 80 |
| Burden  I have not found the Tasty School too stressful. | 22 | 17 | 61 |
| Opportunity Costs  The Tasty School has not taken too much time away from other activities. | 20 | 27 | 53 |
| Intervention Coherence  It is easy for me to understand how the Tasty School benefits me in my work. | 0 | 14 | 86 |
| It is easy for me to understand how our school benefits from the Tasty School. | 1 | 16 | 83 |
| Self-efficacy  I trust in my abilities to carry out the Tasty School. | 0 | 15 | 85 |
| Perceived Effectiveness  The Tasty School has increased cooperation between school teaching personnel who promote food education. | 6 | 19 | 75 |
| The Tasty School has increased cooperation with pupils’ parents who promote food education. | 38 | 38 | 24 |
| The Tasty School has increased cooperation with school food services that promote food education. | 15 | 34 | 51 |
| The Tasty School has increased my confidence in my own abilities to carry out food education at school. | 8 | 31 | 61 |
| The Tasty School has helped me to recognize my own role as a food educator at school. | 9 | 25 | 66 |
| The Tasty School has offered me new information. | 8 | 21 | 71 |
| School teaching benefits from the Tasty School. | 3 | 12 | 85 |
| The ideas in the Idea bank have helped me to put food education into practice. | 2 | 8 | 90 |
| The online training has strengthened my role as a food educator at school. | 3 | 46 | 51 |

^a^ The given response options were totally disagree, somewhat disagree, neither agree nor disagree, somewhat agree, and totally agree. In the table, the value “disagree” contains responses given to totally disagree and somewhat disagree, and the value “agree” contains responses given to totally agree and somewhat agree.
